# Neutralizing Antibody Response of Vaccinees to SARS-CoV-2 Variants

**DOI:** 10.3390/vaccines9050517

**Published:** 2021-05-18

**Authors:** Gabriele Anichini, Chiara Terrosi, Gianni Gori Savellini, Claudia Gandolfo, Federico Franchi, Maria Grazia Cusi

**Affiliations:** 1 Virology Unit, Department of Medical Biotechnologies, Siena University Hospital, 53100 Siena, Italy; gabriele.anichini@student.unisi.it (G.A.); chiara.terrosi@unisi.it (C.T.); gianni.gori@unisi.it (G.G.S.); claudia.gandolfo@unisi.it (C.G.); 2 Emergency, Anesthesia and Intensive Care Unit, Department of Medicine, Surgery and Neurosciences, Siena University Hospital, 53100 Siena, Italy; federico.franchi@unisi.it

**Keywords:** SARS-CoV-2, vaccine, variants, neutralizing antibody

## Abstract

Due to their increased transmissibility, three variants of high concern have emerged in the United Kingdom (also known as B.1.1.7 lineage or VOC-202012/01), South Africa (B.1.351 lineage), and Brazil (P1 lineage) with multiple substitutions in the spike protein. Since neutralizing antibodies elicited by vaccination are likely considered as correlates of protection for SARS-CoV-2 infection, it is important to analyze whether vaccinees with mRNA BNT162b2 are equally protected against these emerging SARS-CoV-2 variants. To this aim, we enrolled healthy subjects one month after complete vaccination with Comirnaty and evaluated the neutralizing response against the native Wuhan strain and the emerging B.1.1.7, B.1.351 and P1 lineages, by using the microneutralization assay, currently considered the gold standard test for the evaluation and detection of functional neutralizing antibodies. The most remarkable finding of this study was the significantly lower neutralizing antibody titer against B.1.351 lineage, compared to the wild-type virus. No significant differences were observed with the other two lineages. These findings provide evidence that vaccinated subjects may not be equally protected against all SARS-CoV-2 lineages.

## 1. Introduction

The severe acute respiratory syndrome coronavirus 2 (SARS-CoV-2) circulating in the human population has acquired multiple mutations, particularly in the spike protein, necessary to bind to the cell-surface angiotensin-converting enzyme 2 (ACE2) receptor. The same viral protein has been targeted for vaccine development and therapeutic antibody interventions [1,2]. On 11 December 2020, the United States of America (U.S.A) Food and Drug Administration issued the first emergency use authorization (EUA) to Pfizer BioNTech (Pfizer Inc., New York, NY, USA)) to use their COVID-19 mRNA BNT162b2 vaccine Comirnaty. It showed a high efficacy (95%) in preventing symptomatic infections in individuals aged 16 years and older [1]. The emergency use authorization allowed the Pfizer-BioNTech COVID-19 vaccine to be distributed with a two-dose formulation, 21 days apart. This formulation is able to induce a strong specific neutralizing antibody response against the wild-type strain [3,4]. Meanwhile, due to their increased transmissibility, three variants of high concern have emerged in the United Kingdom (also known as B.1.1.7 lineage or VOC-202012/01), South Africa (B.1.351 lineage), and Brazil (P1 lineage) with multiple substitutions in the spike protein, including the N-terminal domain (NTD) and the receptor binding domain (RBD). Shared by all the three variants and located in the RBD, the N501Y substitution appears to be highly relevant to increase the affinity to ACE2 [5]. Other significant variations in the UK strain are the amino acid 69 and 70 deletion (Δ69/70) and D614G substitution, which affects higher infectivity and transmissibility [6]. Differently from the B.1.1.7 lineage, P1 and B.1.351 contain the E484K substitution, which is also located in the viral RBD and is known to confer resistance to several monoclonal antibodies [7,8]. Moreover, both P1 and B.1.351 lineages are, respectively, characterized by the K417N and K417T substitutions, which interfere with the neutralizing activity of antibody response [9]. Since neutralizing antibodies elicited by vaccination are likely considered as correlates of protection from SARS-CoV-2 infection, it is important to analyze whether vaccinees with mRNA BNT162b2 are equally protected against these emerging SARS-CoV-2 variants. To this aim, we enrolled healthy subjects one month after complete vaccination with Comirnaty and evaluated the neutralizing response against the native Wuhan strain and the emerging B.1.1.7, B.1.351 and P1 lineages, by using the microneutralization assay (MN). Currently considered as the gold standard test, MN is the most specific and sensitive serological assay when evaluating and detecting functional neutralizing antibodies [10].

## 2. Materials and Methods

### 2.1. Study Design and Participants

In this current observational cohort study, we enrolled 60 healthcare workers (HCWs) from ‘Santa Maria alle Scotte’ University Hospital in Siena, aged 25 to 65 (mean age 45.8; CI 95% 42.9–48.7), 22 males (36.7%; mean age 45.1 years, CI 95% 40.1–50.2) and 38 females (63.3%; mean age 45.6 years, CI 95% 42.0–49.2. They had been subjected to periodical control (every 2 weeks) by molecular testing for SARS-CoV-2 virus with nasopharyngeal swab and had never been infected. All subjects were vaccinated with two doses of BioNTech COVID-19 vaccine (Pfizer Inc.). In order to evaluate the humoral response induced by the vaccine, a blood sample was drawn thirty days after the second dose of vaccine. All subjects were tested for the presence of neutralizing antibodies against the wild-type and three variant lineages of SARS-CoV-2: B.1.1.7 lineage (VOC-202012/01), South Africa (B.1.351 lineage), and Brazil (P1 lineage) by microneutralization test. This research was carried out according to the principles of the Helsinki declaration, with reference to BIOBANK-MIU-2010 document approved by the Ethics Committee with amendment No. 1, on 17 February 2020. Prior to participating in this study, all subjects signed a written informed consent.

### 2.2. Cells and Viruses

Vero E6 cells were grown as a monolayer in Dulbecco’s modified Eagle’s medium (DMEM) (Euroclone, Milan, Italy) supplemented with 100U/mL penicillin/streptomycin (Euroclone) and 5% heat-inactivated fetal calf serum (FCS) (Euroclone) at 37 °C in a humidified 5% CO_2_ atmosphere. SARS-CoV-2 wild-type (SARS-CoV-2/human/ITA/Siena-1/2020; GenBank: MT531537.2), B.1.1.7 (GSAID EPI_ISL_1163688), B.1.351 (GSAID EPI_ISL_1163689) and P.1 (GSAID EPI_ISL_1163690) strains were isolated from clinical swabs and propagated on Vero cells until a cytopathic effect (CPE) appeared. Viral stocks were prepared, titrated on Vero cells and stored at −80 °C for long term.

### 2.3. SARS-CoV-2 Microneutralization Test

**Figure 1:**
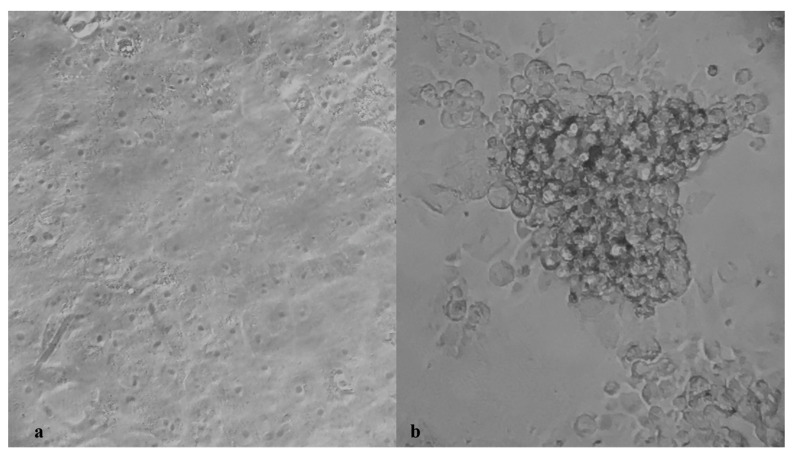
Viral cytopathic effect (CPE) (**b**) observed in cultured Vero E6 cells after infection with 100 TCID of the SARS-CoV-2 strain. (**a**) shows the uninfected control cells. (Magnification 400×).

SARS-CoV-2 virus neutralization assay was carried out on Vero E6 cells in a 96-well microplate. Twenty-five microliters of two-fold serial dilutions (1:8 to 1:1024) of sera samples were added to an equal volume of the wild-type (SARS-CoV-2/human/ITA/Siena-1/2020; GenBank: MT531537.2), B.1.1.7 (GSAID EPI_ISL_1163688), B.1.351 (GSAID EPI_ISL_1163689) and P.1 (GSAID EPI_ISL_1163690) SARS-CoV-2 strains containing 100 TCID_50_ in four replicates and incubated for 90 min at 37 °C. Finally, 50 μL of Vero E6 cells suspension (2 × 10^5^ cells/ml) prepared in complete DMEM were added to each well. After incubation at 37 °C, cultures were daily examined for the presence of CPE under microscope (Olympus IX51, Olympus Corporation, Tokyo, Japan) by the same observer (Figure 1) [11]. The 50% end point geometric mean titer (GMT) was calculated using the Reed−Muench method [12]. A positive and negative control serum were included in each assay. All neutralization assays were performed in duplicate. The test was conducted in a BSL3 lab.

In addition, a regression analysis of the neutralizing antibodies of the study subjects was assessed thirty days after vaccination, according to subject age.

### 2.4. Statistical Analysis

Age differences and neutralizing GMTs were evaluated and statistical significances were assessed with the Mann−Whitney−Wilcoxon test. Results were considered statistically significant at *p* < 0.05. A 95% confidence interval (CI 95%) has been calculated and reported for each variable. Regression analysis of the neutralizing antibodies according to the participants’ age was assessed. All analyses were performed by using Graph Pad Prism 7.0 software (GraphPad Software, San Diego, CA, USA)).

## 3. Results

### 3.1. Study Group

We analyzed sera from 60 healthcare workers (HCWs), aged 25 to 65 (mean age 45.8; CI 95% 42.9–48.7), 22 males (36.7%; mean age 45.1 years, CI 95% 40.1–50.2) and 38 females (63.3%; mean age 45.6 years, CI 95% 42.0–49.2), who had never been infected with SARS-CoV-2 virus.

All subjects were found to be positive for specific IgG antibodies one month after the second dose of vaccine.

### 3.2. Neutralizing Antibody Response against SARS-CoV-2 Variants

**Figure 2:**
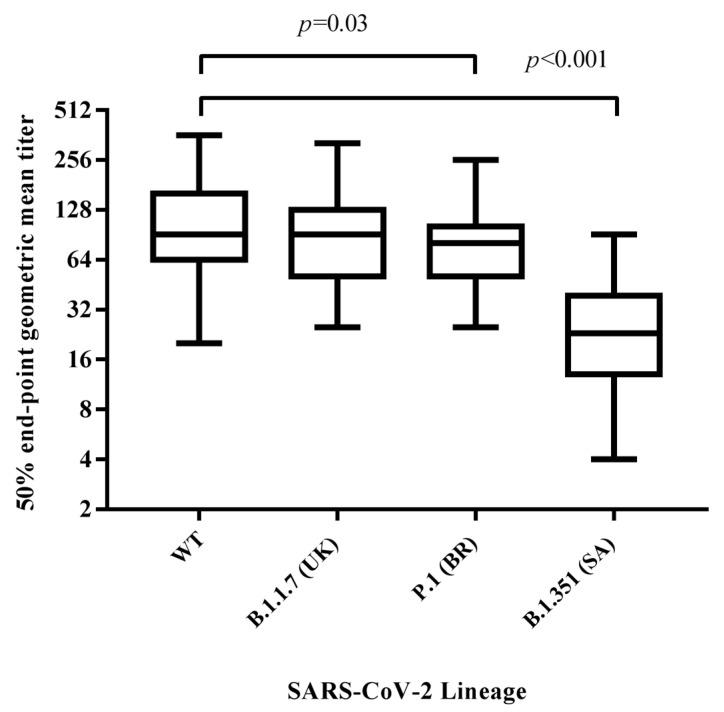
Differences in neutralizing antibody titers among vaccinated subjects against the wild-type, B.1.1.7, B.1.351, and P.1 SARS-CoV-2 lineages thirty days after receiving the second dose of vaccine. Results are reported in the box−whiskers plots as GMTs and upper and lower quartiles. GMT, geometric mean titer.

To better characterize the humoral response induced by vaccination, neutralizing antibodies against the wild-type virus and the three variant lineages were investigated. Only one subject, who tested weak positive for IgG by the chemiluminescent assay (CMIA), did not develop a neutralizing response against all four lineages. With regard to the remaining 59 subjects, the neutralizing antibody titers elicited against the wild-type strain (GMT = 95.6, CI 95% 79.1–112.0) showed a slight decrease (1.2 fold, *p* = 0.03) versus P1 lineage (GMT = 78.5, CI 95% 76.6–100.0) and a significant decrease (4.2 fold, *p* < 0.001) to the B.1.351 lineage (GMT = 22.8, CI 95% 17.8–27.9). No significant differences were found in comparison with the B.1.1.7 (UK) lineage (GMT = 89.1, CI 95% 73.6–105.0) (Figure 2).

### 3.3. Regression Analysis of Neutralizing Titers According to Subject Age

**Figure 3:**
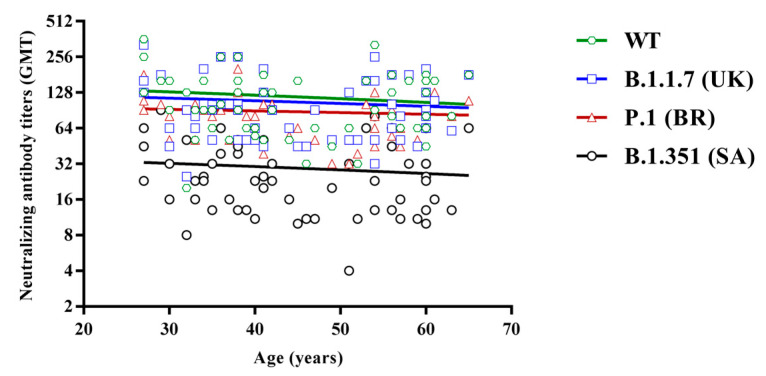
Regression analysis of neutralizing antibody titers against the wild-type (green, R2 = −0.12), B.1.1.7 (blue, R2 = −0.09), P.1 (red, R2 = −0.07) and B.1.351 lineages (black, R2 = −0.11), according to subjects’ age.

Moreover, neutralizing titers were analyzed considering the subjects’ age. To this aim, they were divided into two groups according to their age: 25–45 and 46–65 years. No relevant differences were found between the two groups, either in circulating IgG values or in neutralizing GMTs, to all four lineages, although a very slight decrease was observed with increasing age (Figure 3).

## 4. Discussion

In this study, we evaluated the neutralizing activity against recently emerging variants of SARS-CoV-2 in subjects vaccinated with two doses of BioNTech COVID-19 vaccine.

Instead of pseudoviruses, we used clinical isolates of variants in the neutralization assay, thus providing a relevant method to assess the viral sensitivity to neutralizing antibodies in the presence of all SARS-CoV-2 antigen epitopes. We preferred to perform this procedure, considered as the gold standard, because the neutralizing antibody titers against SARS-CoV-2 variants are so different, depending on the test used [13]. We realized that, although different, the antibody response trend was similar. Indeed, the results reported in this study are similar to others that used different methods to measure the efficacy of the vaccine against variants after about a month [4,13,14,15,16]. However, despite the unquestionable scientific relevance of different methods that may be carried out, a uniformity in reporting absolute titer values in International Standard Units is desirable, in order to avoid diverse interpretations of the antibody trend several months after vaccination.

The B.1.351 variant is currently of greater concern, being less sensitive to sera from immunized people [14,15,16,17]. The most remarkable finding of this study is the significantly lower neutralizing antibody titer against B.1.351 lineage, compared to the Wuhan-Hu-1 virus. Indeed, we found that B.1.351 was approximately 4-fold less sensitive to neutralization by vaccinees’ sera, whereas P.1 variant was only 1.2-fold less sensitive. On the other hand, the GMT against B.1.1.7 was similar to that obtained against the wild type virus (89.1 vs. 95.6). Moreover, the neutralizing response appeared reduced, particularly against variants carrying the K417N/T and E484K mutations. Both of them are located in the receptor binding domain (RDB) of the spike protein and can mediate antibody escape. However, since the cross-reactivity to B.1.351 of antibody response appeared very low, other mutations were likely to be involved, particularly affecting the N-terminal domain of the viral spike [18,19,20]. These findings provide evidence that vaccinated subjects may not be equally protected against all SARS-CoV-2 lineages. Therefore, considering the up to four-fold decrease in the GMT tested against B.1.351 lineage, it is likely that only people having a high antibody titer could be protected, although a threshold of protection still needs to be defined.

## Data Availability

The authors declare that the data supporting the findings of this study are available within the paper or available from the corresponding author(s) upon reasonable request.
